# Effects of Larch Woolly Adelgid Infestation on Morphological, Histological and Allelochemical Traits of European Larch Needles

**DOI:** 10.3390/insects15120938

**Published:** 2024-11-28

**Authors:** Katarzyna Dancewicz, Bożena Kordan, Marta Damszel, Iwona Sergiel, Magdalena Biesaga, Joanna Mroczek, Beata Gabryś

**Affiliations:** 1 Department of Botany and Ecology, University of Zielona Góra, Szafrana 1, 65-516 Zielona Góra, Poland; b.gabrys@wnb.uz.zgora.pl; 2 Department of Entomology, Phytopathology and Molecular Diagnostics, University of Warmia and Mazury, Prawocheńskiego 17, 10-720 Olsztyn, Poland; bozena.kordan@uwm.edu.pl (B.K.); marta.damszel@uwm.edu.pl (M.D.); 3 Department of Biotechnology, University of Zielona Góra, Szafrana 1, 65-516 Zielona Góra, Poland; i.sergiel@wnb.uz.zgora.pl; 4 Department of Chemistry, University of Warsaw, Pasteura 1, 02-093 Warszawa, Poland; mbiesaga@chem.uw.edu.pl (M.B.); joannagasik93@gmail.com (J.M.)

**Keywords:** *Adelges laricis*, *Larix decidua*, conifer defense, phenolic compounds, flavonoids

## Abstract

The study was carried out to assess the effect of the larch wooly adelgid *Adelges laricis* Vallot (Hemiptera: Adelgidae) infestation on its secondary host, the European larch *Larix decidua* Mill. Morphology and anatomy of adelgid-infested needles, and content of defense phenolic compounds including individual flavonoids isorhamnetin, kaempferol, quercetin, rutin, catechin, epicatechin, apigenin, ampelopsin and taxifolin, were analyzed. The amount of total phenols in needles from adelgid-infested twigs of *L. decidua* increased following the development of the *A. laricis* population, from the end of April until the end of June. The most abundant among flavonoids were the flavanols, mainly catechin and epicatechin, which predominated in the larch needles during the whole period of adelgid infestation. The content of catechin and epicatechin increased following the increase in the adelgid population number. An increase in content occurred also in flavanonols ampelopsin and taxifolin, while the content of the flavonol kaempferol decreased as the population number of *A. laricis* increased. The analysis of the anatomical structure of needles showed changes in the shape of the needles, the presence of a thicker layer of epicuticular waxes, and a higher number of mesophyll layers as a result of adelgid feeding.

## Simple Summary

Plant defense mechanisms can be divided into two categories: structural and chemical. Using morphological and biochemical analysis methods, we investigated the effect of the larch wooly adelgid *Adelges laricis* Vallot infestation on its secondary host, the European larch *Larix decidua* Mill. The population of the larch wooly adelgid developed from the end of April until the end of June, with the peak number in the first days of May. Anatomical examination of larch needles revealed the altered shape of the needles, the presence of a thicker layer of wax covering the epidermis and an increase in the number of layers of mesophyll as the results of adelgid feeding. The increased amounts of total phenols and individual flavonoids in adelgid-infested needles corresponded with the increase in the larch wooly adelgid numbers. The most abundant flavonoids were catechin and epicatechin, which predominated in larch needles collected at successive phases of adelgid infestation and their content increased along the increase in adelgid infestation. In contrast, the content of kaempferol decreased. These results greatly expand the knowledge on the anatomical and biochemical mechanisms of adelgid-conifer interaction.

## 1. Introduction

The genus larch *Larix* Mill. (Pinaceae) comprises deciduous conifer species that occur in Eurasia and North America [1,2,3]. The European larch *Larix decidua* Mill. is native to the mountains of central Europe, the Alps and Carpathians, and mainly grows in mixed stands with other conifer species, the Norway spruce *Picea abies* (L.) H. Karst, stone pine *Pinus cembra* L., Swiss pine *Pinus mugo* Turra, silver fir *Abies alba* Mill., or deciduous species such as the European beech *Fagus sylvatica* L. (Fagaceae) [4,5,6]. It can also occur in pure stands or is cultivated as an ornamental plant in parks and urban areas outside its natural ranges [5,7,8]. The European larch is also a commercially valuable tree species; therefore the spectrum of its insect pests, which belong mainly to Coleoptera, Lepidoptera, Diptera, and Hemiptera, is quite well known [9,10,11,12,13,14]. Within Hemiptera, the adelgids (Adelgidae) are serious pests of conifer trees [15,16,17,18,19,20,21,22]. Similar to the closely related groups of Hemiptera, the phylloxerans (Phylloxeridae) and aphids (Aphididae), the adelgids possess sucking–piercing mouthparts and, besides transmitting plant virus diseases, directly damage their host plants by removing plant sap and/or inducing galls [18,19,20,21,22]. The larch woolly adelgid *Adelges laricis* Vallot (Hemiptera: Adelgidae) is an important pest of Norway spruce and European larch [23,24,25,26]. In spruce, *A. laricis* induces galls, while in larch trees, the feeding by *A. laricis* causes chlorosis, stunting and distortion of the needles [12,20]. In addition, the larvae and adults of the parthenogenetic generations produce copious wax and honeydew, so, if the infestation is heavy, the larch tree appears bluish in color [11,14,20,24]. However, the effect of *A. laricis* infestation on the morphological and anatomical structure of the needles has never been described in detail.

During the long history of coevolution of plants and phytophagous organisms, plants generated different mechanisms that protect them against herbivores or allow the regeneration of damaged parts. Plant resistance to herbivores is a combination of constitutive (‘static’—the genetically inherited qualities that are permanently present in plants) and induced (‘active’—the qualities that are expressed in response to abiotic and biotic stresses, including herbivore infestation) structural and biochemical defense mechanisms [27,28,29,30,31,32].

Plant structures, such as leaf surface wax, thorns or trichomes, and cell wall thickness and lignification, form the first physical barrier to feeding by herbivores, including hemipterans, and are important components of constitutive and induced defenses [27,28,33,34,35] in conifers. The constitutive structural defenses include also the outer and inner bark. The outer bark consists mostly of lignified and suberized cells while the inner bark and secondary phloem contains a variety of defense-related cell types such as heavily lignified sclerenchyma and sclereids, and cells containing calcium oxalate crystals [27,28,31,36,37]. The induced defense mechanisms in conifers involve changes in cell division and differentiation, leading to the formation of traumatic resin ducts in the xylem or phloem [37,38]. In addition, inducible defense responses include activation of the existing and production of new polyphenolic parenchyma cells and wound periderm formation or an activation of the hypersensitive response [28,37,38].

The biochemical constitutive and inducible defense mechanisms of plants depend on the presence of allelochemicals, a group of secondary plant metabolites that are able to modify negatively the behavior and development of herbivores, including phytophagous insects [27,28,29,30,31]. Generally, the constitutive level of allelochemicals determines the suitability of the plant species for colonization and exploitation by herbivores and thus governs host preferences and acceptability. The attack by pests and pathogens triggers a signaling cascade involving the jasmonic acid and salicylic acid pathways, which results in an increased synthesis and storage of the defense chemicals [27,28,36,38,39,40,41,42,43]. In conifers, the production of oleoresin and phenolic compounds is the most important chemical defense mechanism against herbivores [42]. The resin, containing numerous terpenoids, is produced by special secretory tissues and stored in a network of resin ducts in the wood, bark, roots and needles [28,38,43]. Phenolic compounds are produced in specialized ray cells and polyphenolic parenchyma cells and stored abundantly in bark, roots and needles [28,31,44]. The phenolic compounds such as stilbenes, flavonoids, lignans and tannins are the major class of inducible defense compounds in conifers, as in many woody species [28,38,45,46].

Among phenolics, the flavonoids play the broadest variety of functions, including their roles as UV protectants, antioxidants, signal molecules, allelopathic compounds, phytoalexins, and anti-herbivore chemical defense factors [46,47,48,49]. As anti-herbivore agents, flavonoids affect the behavior, development and growth of a number of insects and play at least a partial role in plant resistance to several pests [46,50,51,52,53,54]. Although the phenolics and especially the flavonoids are considered major defense compounds against herbivores in conifers [28,29], knowledge of the effect of individual compounds on herbivorous insects that infest Pinaceae is limited. A bioassay revealed that the dihydroflavonol taxifolin and the flavan-3-ol catechin from Norway spruce negatively affected the bark beetle *Ips typographus* (L.) (Coleoptera: Curculionidae) [55]. Only limited information is available about phenolic compounds in Pinaceae as biomarkers responding to climatic and environmental changes [56,57]. Several studies have shown the constitutive and induced content of flavonoids such as quercetin, taxifolin, ampelopsin, naringenin, catechin, epicatechin, isorhamnetin, and kaempferol in the needles, stems and roots of coniferous trees [10,58,59,60,61,62,63]. However, precise information on the content of these compounds in response to herbivore infestation of conifers is scarce. In particular, no data on the content of phenolic compounds in any species of *Larix* in response to adelgid infestation, including *A. laricis*, are available.

The purpose of the present study was to fill some gaps in the knowledge on the *A. laricis*—*L. decidua* relationship. In particular, our goals were to (i) determine the population dynamics of the larch wooly adelgid on its secondary host, the European larch; (ii) analyze the content of total phenolics and selected flavonoids in adelgid-infested larch needles at different stages of adelgid population development; and (iii) examine the morphology and anatomy of adelgid-infested and uninfested needles. Considering the existing knowledge on herbivore—plant phenolic relationships, we hypothesized that adelgid infestation induces changes in the content of total phenols and individual flavonoids in larch needles and that the amounts of these allelochemicals vary depending on the course of adelgid population development on *L. decidua*. We also hypothesized that adelgid infestation induces changes in larch needle external and internal morphology.

## 2. Materials and Methods

### 2.1. Research Locality

The study was carried out in the garden of the Department of Botany and Ecology, University of Zielona Góra, Monte Cassino 21b, Zielona Góra (51.9323213, 15.4893333; Lubuskie Province, Poland).

### 2.2. Population Dynamics and Population Structure of Adelges laricis on Larix decidua

The object of study was a European larch *Larix decidua* Mill. tree naturally infested by *Adelges laricis.* No protective insecticide treatments were applied to the tree.

The monitoring of the occurrence of *A. laricis* on *L. decidua* commenced in the beginning of April and was terminated in the last week of June 2011, which coincided with the appearance of the first adelgid individuals on the larch tree and the decline of the population, respectively. The exact dates of observations were 9, 20, 29 April, 10, 20, 30 May and 10, 19, 30 June. For the monitoring of the number and age structure of the *A. laricis* population, 25 twigs (five 25 cm long twigs on five sides of the tree), approximately 1.6 m from the ground, were randomly selected and labeled. The twigs were inspected in the morning hours from 8.00 to 11.00. The timing for collection of the field data was based on knowledge of the diurnal rhythms of aphids [64]. As no information on adelgid diurnal activity is available, we applied the information on their closest relatives, the aphids. At each inspection time, on the dates given earlier, the number of individuals and the developmental stages of *A. laricis* were recorded and identified according to Carter [65] and Blackman and Eastop [66]. The following developmental stages of *A. laricis* were recorded: *exulis sistens* females (ESF = deriving from the nymphs overwintering at the dwarf stem bases), *exulis progrediens* crawlers (EPC = 1st instar *progrediens* nymphs on needles), *exulis progrediens* woolly nymphs and/or adults (EPW = advanced nymphs and adult females producing the waxy ‘wool’ that covers their bodies), and winged *sexupara* females (SW = winged adults without ‘wool’).

The samples of individual developmental stages of *A. laricis* were collected for taxonomic evaluation and photographic documentation. The material was examined under a stereoscopic microscope Olympus Camedia C-3030 ZOOM digital camera (Olympus Polska sp. z.o.o, Warszawa, Poland) paired with Olympus DP-Soft 3.1 PC software (Olympus Polska sp. z.o.o, Warszawa, Poland) in the laboratory of Department of Botany and Ecology, University of Zielona Góra, Poland.

The meteorological data were obtained from IMGW PIB (Instytut Meteorologii i Gospodarki Wodnej Państwowy Instytut Badawczy = Institute of Meteorology and Water Management, National Research Institute; Podleśna 61, 01-673 Warszawa, Poland).

### 2.3. Chemical Analysis

In the present study, the contents of total phenols and individual flavonoids ampelopsin, apigenin, catechin, daidzein, epicatechin, glycitein, hesperetin, hesperidin, isorhamnetin, kaempferol, luteolin, naryngin, quercetin, resveratrol, rutin, and taxifolin were determined in larch needles. The selection of the compounds for analysis was based on literature data that reported major flavonoid constituents of needles from different species of conifers, including the larch [60,61,62,67,68,69].

For chemical analyses, the plant material was collected from the same European larch tree on which the larch wooly adelgid population was monitored. The adelgid-infested larch twigs (five 15–20 cm long twigs from five sides of the tree; n = 25) were collected at random from branches approximately 1.5 m above the ground, every third branch, similarly to the procedure by Dancewicz et al. [70]. The material was collected three times during the *A. laricis* population occurrence, i.e., on 20th April, 10th May and 19th June. The twigs were transported to the laboratory, where they were cleaned of the adelgids and maintained at +4 °C until the sample preparation procedures. From each twig, a sample of needles was taken and individually cleaned with distilled water on a glass plate. The needles were dried (+60 °C, laboratory dryer Wamed SUP-65 (WAMED Wytwórnia Aparatury Medycznej s.s.p., Warszawa, Poland) and kept in darkness at +4 °C temperature until the analysis.

The dried larch needles (1.2 g) were homogenized in an aqueous ethanol solution (80%) using a Diax 900 homogenizer. The resulting suspension was centrifuged (12,000 rpm, 10 min), the supernatant solution was collected in a graduated flask, and the pellet was reconditioned. This operation was repeated three times, and the obtained extracts were combined. The homogenization procedure in combination with the extraction was carried out in such a way that the final volume of the extract was 100 mL. Resulting ethanolic extracts containing phenolic compounds were analyzed by UV-VIS spectrophotometer (UV-VIS Shimadzu Corporation 2450, Kyoto, Japan). For chromatographic analyses, 10 mL was taken from the prepared ethanol extracts and evaporated to dryness in a rotary evaporator under reduced pressure at 40 °C. The dry extracts were dissolved in 100% methanol to a volume of 1 mL.

#### 2.3.1. Analysis of Total Phenols

Colorimetric reactions are widely used in the UV/VIS spectrophotometric method, which is easy to perform, rapid and applicable in routine laboratory use, and low-cost. Polyphenols in plant extracts react with specific redox reagents (Folin–Ciocalteu reagent) to form a blue complex that can be quantified by visible-light spectrophotometry. The reaction forms a blue chromophore constituted by a phosphotungsticphosphomolybde- num complex where the maximum absorption of the chromophores depends on the alkaline solution and the concentration of phenolic compounds.

Folin–Ciocalteu reagent, ethanol and anhydrous sodium carbonate were supplied by Chempur (Chempur, Piekary Śląskie, Poland). Gallic acid was purchased from Sigma-Aldrich (Poznań, Poland). Stock standard solutions of gallic acid (20 mg/L) were prepared by dissolving appropriate amounts of solid reagents in ethanol.

Mixed working standard solutions of gallic acid at 2.0; 4.0; 6.0; 8.0 and 10 mg/L were prepared by appropriate dilutions of stock standard solutions. Then, 0.2 mL of each working standard solution, 0.5 mL of Folin–Ciocalteu reagent and, after 3 min, 2.0 mL anhydrous sodium carbonate (20% *w*/*v*) were transferred to a 10 mL volumetric flask. After 30 min, the absorbance corresponding to total polyphenols was measured at 760 nm with a spectrophotometer Shimadzu UV-2450 (UV-VIS Shimadzu Corporation 2450 Kyoto, Japan). Ethanol was used as a blank.

Then, 0.2 mL of each larch extract, 0.5 mL of Folin–Ciocalteu reagent and, after 3 min, 2.0 mL anhydrous sodium carbonate (20% *w*/*v*) were transferred to a 10 mL volumetric flask. After 30 min, the absorbance corresponding to total polyphenols was measured at 760 nm with a spectrophotometer Shimadzu UV-2450. All assays were conducted in triplicate. A dose–response linear regression (R2 = 0.991) was generated by using the gallic acid standard absorbance, and the levels in the samples were expressed as gallic acid equivalents (µg/g).

#### 2.3.2. Analysis of Flavonoids

Individual pure flavonoids (ampelopsin, apigenin, catechin, daidzein, epicatechin, glycitein, hesperetin, hesperidin, isorhamnetin, kaempferol, luteolin, naryngin, quercetin, resveratrol, rutin, taxifolin) were purchased from Sigma-Aldrich (Poland). High-performance liquid chromatography (HPLC) gradient grade methanol and acetonitrile were supplied by Merck (Darmstadt, Germany). Formic acid was purchased from Sigma-Aldrich (Poland). Stock standard solutions of single phenolic compounds (50 mg/L) were prepared by dissolving appropriate amounts of solid reagents in methanol. Mixed working standard solutions of phenolic compounds at 20, 10, 5, 2.5 and 1 mg/L or lower concentrations were prepared by appropriate dilutions of stock standard solutions.

The chromatographic analysis was carried out with a Shimadzu LC system, comprising an LC20-AD binary pump, a DGU-20A5 degasser, a CTO-20AC column oven and a SIL-20AC autosampler, connected to a 3200 QTRAP hybrid triple quadrupole (Applied Biosystem, MDS SCIEX, Applied Biosystem, MDS SCIEX, Foster City, CA, USA) with electrospray ionization source (ESI) operated in negative-ion mode. Phenolic compounds were separated on a Phenomenex Luna C-18 column (100 × 2.0 mm × 3.0 µm) with a pre-column, both maintained at 30 °C. A 7.4 mmol/L solution of formic acid (pH 2.8, eluent A) and acetonitrile (eluent B) was used. The mobile phase was delivered at 0.2 mL/min in linear gradient mode as follows: 0–2 min 10% B, 30 min 60% B, 40 min 100% B, 55 min 10% B. Phenolic compounds were identified by comparing their retention times and m/z values of precursor and resulting fragmentation product ions in their MS and MS/MS spectra, respectively, to those obtained for the respective standard solutions analyzed under the same conditions. The quantification of phenolic compounds was performed using calibration curves obtained in the single reaction mode (SRM) [71,72].

In the concentration range used for the studied phenolic compounds, the response of the system was found to be linear, with determination coefficients (R^2^) better than 0.998. The reproducibility of signals obtained with LC–ESI-MS/MS was evaluated by seven consecutive injections of standard solutions (1 mg/L) and expressed as relative standard deviations (RSDs). RSD values at the levels of intra-day and inter-day precision were better than 2% and 5%, respectively.

### 2.4. Needle Anatomy

For histological analysis, the larch needles were collected on 10th May, which coincided with the peak of the adelgid population. The adelgid-free needles without deformations and adelgid-infested needles with visible deformations were selected. For anatomical examination, 0.5 mm thick needle sections at mid-length of the adelgid-free needles and at the deformation spot of adelgid-infested needles were cut and fixed for 24 h in Karmovsky’s fixative containing 10% paraformaldehyde and 25% glutaraldehyde in 0.2 M phosphate buffer (pH 7.2). The fixed material was rinsed three times (15 min each time) in 0.1 M phosphate buffer (pH 7.4), dehydrated in a graded ethanol series (for 10 min in 30%, 50%, 70%, and 96% ethanol and 2 × 10 min in 99.8% ethanol), and placed in 100% polypropylene oxide (2 × 10 min). The slices were then placed overnight in a 1:1 mixture of epon resin (5.25 mL Poly/Bed 812, 3.25 mL DDSA, 0.75 mL MNA, 0.175 DMP) and propyl oxide. Next day, the plant samples were embedded in 100% epon resin. Transverse slices (0.5 μm) of the needles were obtained using a rotary automatic microtome Leica HistoCore Nanocut R (Leica Biosystems Nussloch GmbH, Nussloch, Germany). Slices were stained with toluidine blue, mounted on slides and observed under light microscope Carl Zeiss Axio Imager 2 (Carl Zeiss Microscopy GMbH, Jena, Germany) coupled with Zeiss AxioCam ERc 5s and ZEN Lite computer program (Carl Zeiss Microscopy GMbH, Jena, Germany; Carl Zeiss Microspoy GMbH, Göttingen, Germany)at 400× magnification.

### 2.5. Statistical Analysis

The statistical analysis of *A. laricis* population development data included descriptive statistics (mean ± SE) of all variables.

The results of chemical analyses are presented as means of triplicates for each variable. Data thus obtained were examined using the analysis of variance ANOVA (*p* = 0.05) and multiple comparison procedure (post hoc Newman–Keuls test) [73]. All statistical calculations were performed using StatSoft, Inc. STATISTICA (data analysis software system), version 13.3., www.statsoft.com.

## 3. Results

### 3.1. Occurrence of Adelges laricis on Larix decidua

**Figure 1:**
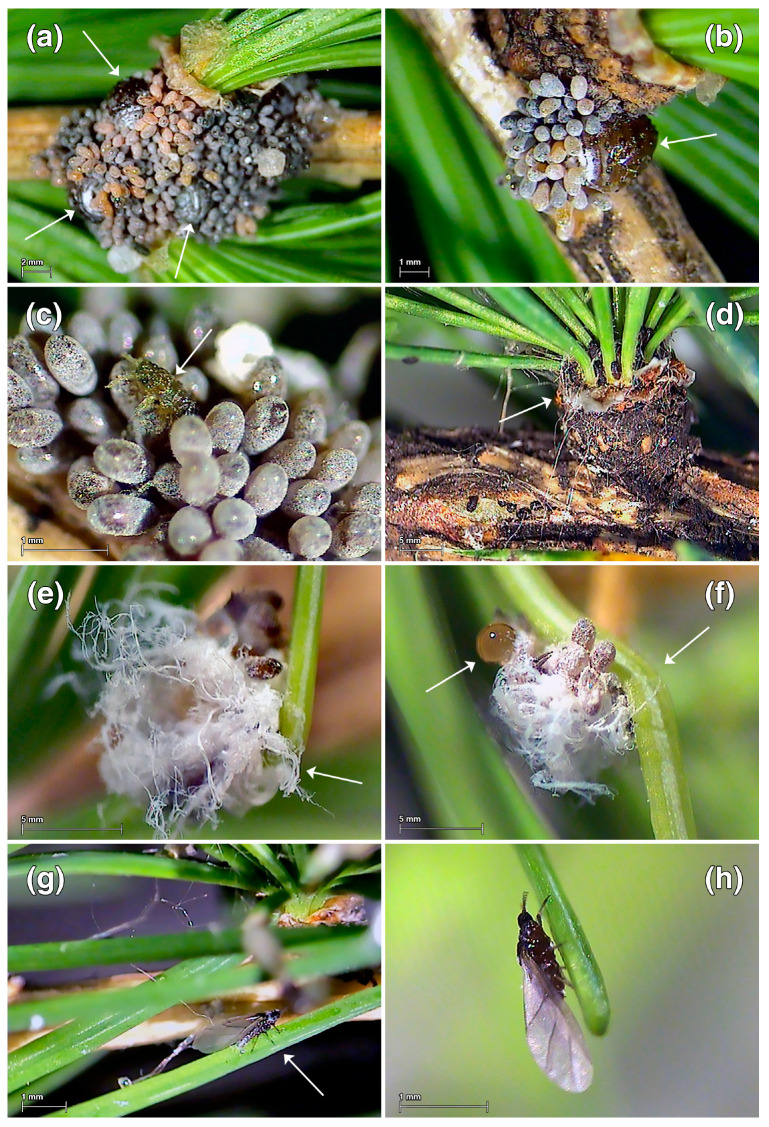
Parthenogenetic developmental stages of *Adelges laricis* generations on *Larix decidua*. (**a**) ESF—*Exulis sistens* females at dwarf stem base with large clutch of eggs; the arrows point to the females. (**b**) ESF—*Exulis sistens* female at dwarf stem base with egg masses; the arrow points to the back of the body and glandular body surface. (**c**) EPC—*Exulis progrediens* mobile nymph (crawler)—1st instar nymph after hatching; the arrow points to the crawler. (**d**) EPC—*Exulis progrediens* mobile nymphs (crawlers) at dwarf stem base. (**e**,**f**) EPW—*Exulis progrediens* ‘woolly’ (wax-covered) sessile nymphs or adults with eggs and honeydew on larch needle; the arrows point to the deformation of needles and the droplet of honeydew. (**g**,**h**) SW—Winged *sexupara* females on larch needle. Images were obtained using an Olympus Camedia C-3030 ZOOM digital camera paired with Olympus DP-Soft 3.1 PC software.

The developmental stages of two parthenogenetic generations (*exules* and *sexuparae*) of *A. laricis* were identified on larch: *exulis sistens* wingless females at dwarf stem bases (ESF), *exulis progrediens* crawlers, which are the mobile 1st instar nymphs on needles (EPC), *exulis progrediens* ‘woolly’ sessile nymphs and ‘woolly’ adults on needles (EPW nymphs and EPW females, respectively) and winged *sexupara* females (SW) (Figure 1a–h). ESF females were brown to purplish black with few wax glands and deposited masses of yellowish to reddish-brown eggs at the dwarf stem bases (Figure 1a,b). The mobile EPC nymphs, which hatched from these eggs, crawled to the needles and settled on them. The crawlers were broadly ovoid to tapered spheroid and yellow to red-brown after hatching (Figure 1c,d). The sessile, late-instar EPW nymphs were dark brown or black, covered with white, waxy ‘wool’ (Figure 1e). The EPW nymphs developed into EPW adult females (wingless ‘woolly’ females) (Figure 1f) that remained on the larch needles, or into SW adults (winged females without ‘wool’) (Figure 1g,h) that became the *sexuparae* that migrated back to spruce trees.

**Figure 2:**
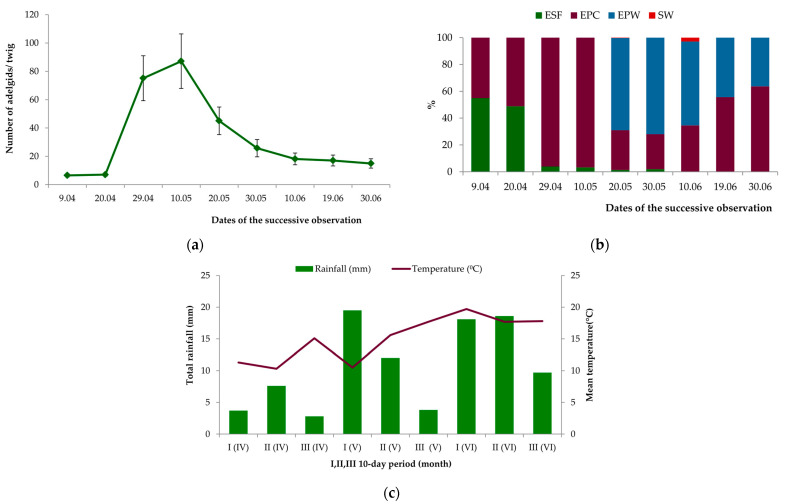
Occurrence of *Adelges laricis* on *Larix decidua.* (**a**) Population dynamics of *A. laricis* on *L. decidua* (mean number of adelgids per larch twig ± SE; n = 25); (**b**) Population structure of *A. laricis* on *L. decidua.* ESF—*exulis sistens* females at dwarf stem base; EPC—*exulis progrediens* crawlers (1st instar mobile nymphs) on needles; EPW—*exulis progrediens* ‘woolly’ sessile nymphs or adults on needles; SW—winged *sexsuparae*.; n = 25. (**c**) Total rainfall and mean temperatures in Zielona Góra in 2011. Arabic numerals denote the 10-day period of a month (I—1–10; II—11–20; III—21–30/31); Arabic numerals in parentheses denote the month (IV—April; V—May; VI—June) (data from IMGW PIB—Instytut Meteorologii i Gospodarki Wodnej, Państwowy Instytut Badawczy = Institute of Meteorology and Water Management, National Research Institute; Podleśna 61, 01-673 Warszawa, Poland).

The first individuals of *A. laricis* were recorded on larch in the beginning of April; 6.6 ± 1.1 adelgids per twig were recorded on average (Figure 2a). The number of adelgids remained relatively low until the 20th of April. At that time, *exulis sistens* females (ESF) and *exulis progrediens* crawlers (EPC) predominated in the age structure of the population (Figure 2b). The size of the larch woolly adelgid population peaked in the first 10-day period of May (87.2 ± 19.3 adelgids per twig) (Figure 2a). The EPCs predominated in the age structure of the population at that time (Figure 2b). After its period of maximum size, the *A. laricis* population rapidly decreased until the end of May. In June, a slow decrease in larch woolly adelgid numbers continued. At that time, the most abundant were EPCs and *exulis progrediens* ‘woolly’ sessile nymphs and ‘woolly’ adults (EPW) (Figure 2b). During the same period, the appearance of winged *sexupara* females (SW) was recorded (Figure 2b). At the end of June, only a few EPC and EPW individuals occurred on larch (15.0 ± 3.3 adelgids per twig) (Figure 2a,b).

The mean daily temperatures and the rainfall in spring and summer, calculated for each 10-day period of observation from the beginning of April until the end of June, were variable and differed among individual months (Figure 2c).

### 3.2. Total Phenols in Needles from Adelges laricis-Infested Twigs of Larix decidua

**Figure 3:**
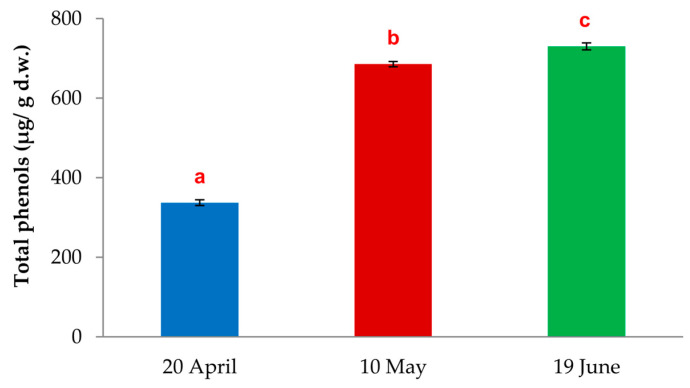
Total phenols in needles from *Adelges laricis*-infested twigs of *Larix decidua* (μg/g d.w.). The sample collection dates: 20th of April (initial phase of adelgid infestation), 10th of May (peak phase of adelgid infestation) and 19th of June (decline phase of adelgid infestation); mean ± SE; n = 3; different letters show significant differences (one-factor ANOVA at *p* = 0.05 and post hoc Newman–Keuls test).

The total phenols content differed significantly among the needles from adelgid-infested twigs of *L. decidua*, which were collected at different sampling times (ANOVA, *p* = 0.0000). The chemical analyses showed the lowest content of total phenols in the needles collected on 20th of April (336.9 ± 4.1 µg/g d.w.). The needles from 10th of May had higher amounts of total phenols (685.5 ± 3.7 µg/g d.w.). The highest content of total phenols was found in the larch needles collected on 19th of June (730.0 ± 5.0 µg/g d.w.) (Figure 3).

### 3.3. Flavonoids in Needles from Adelges laricis-Infested Twigs of Larix decidua

**Figure 4:**
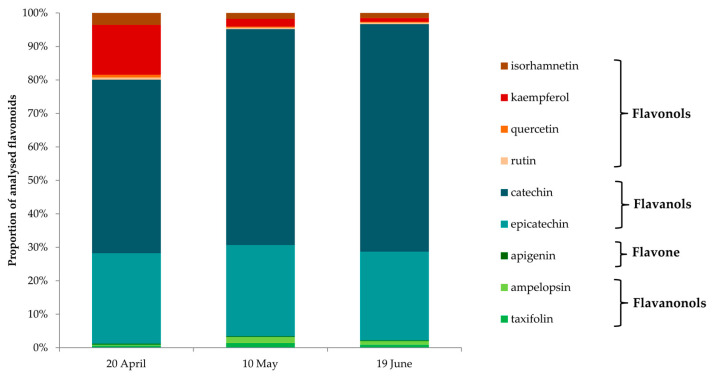
Proportions of flavonoids analyzed in needles from *Adelges laricis*-infested twigs of *Larix decidua.* The sample collection dates: 20th of April (initial phase of adelgid infestation), 10th of May (peak phase of adelgid infestation) and 19th of June (decline phase of adelgid infestation).

**Figure 5:**
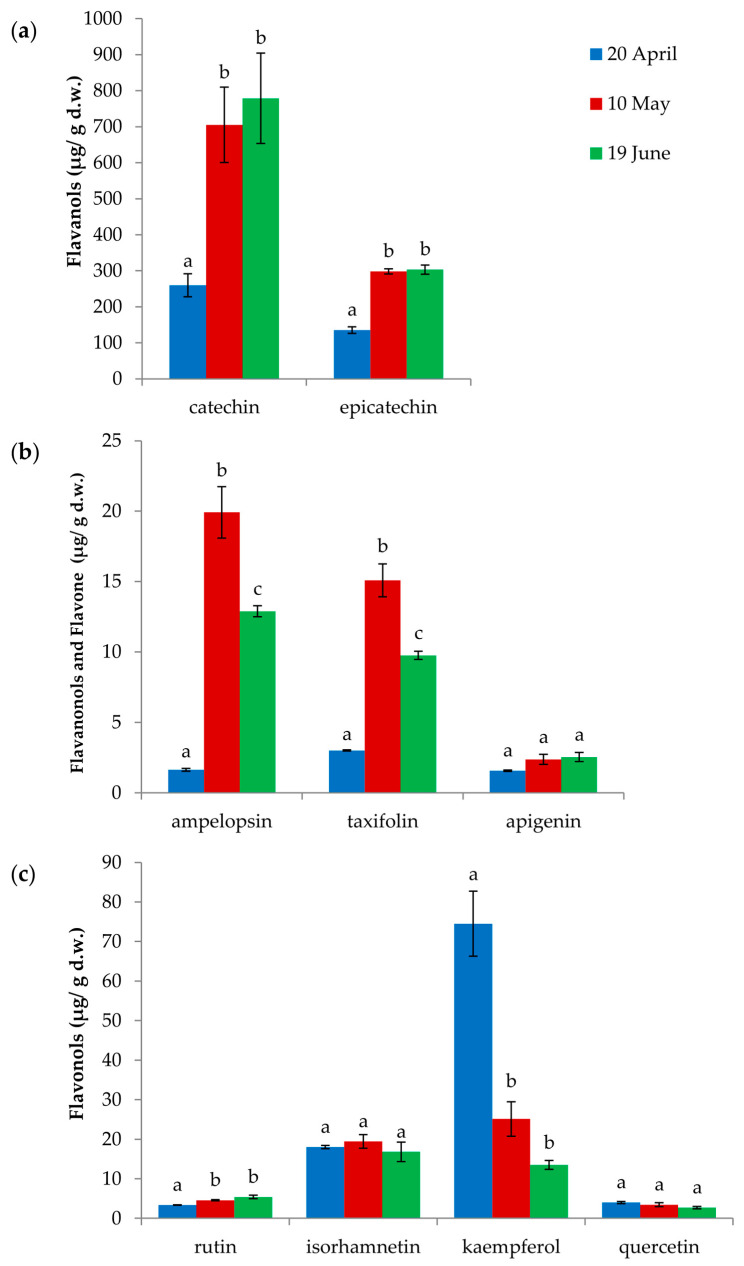
Flavonoids in needles from *Adelges laricis*-infested twigs of *Larix decidua* (μg/g d.w.). (**a**) Flavanols. (**b**) Flavanonols and flavones. (**c**) Flavonols. The sample collection dates: 20th of April (initial phase of adelgid infestation), 10th of May (peak phase of adelgid infestation) and 19th of June (decline phase of adelgid infestation); (mean ± SE; n = 3); different letters show significant differences (one-factor ANOVA at *p* = 0.05 and post hoc Newman–Keuls test).

In the needles from adelgid-infested twigs of *L. decidua*, nine of the analyzed flavonoids were identified: flavonols isorhamnetin, kaempferol, quercetin, rutin; flavanols catechin, epicatechin; flavone apigenin; and flavanolols ampelopsin and taxifolin (Table S1, Figure 4 and Figure 5). The groups of analyzed flavonoids occurred in different amounts and proportions in needles from adelgid-infested twigs of *L. decidua*, which were collected at different sampling times. In all larch needles, the most abundant flavonoids were catechin (51.8%, 64.5% and 68.0% of total flavonoids, respectively, for 20th of April, 10th of May and 19th of June) and epicatechin (27.0%, 27.3% and 26.5% of total flavonoids, respectively, for 20th of April, 10th of May and 19th of June). Moreover, kaempferol accounted for a noteworthy share of the analyzed flavonoids in larch needles collected on 20th of April (14.9% of total flavonoids) (Figure 4).

Statistically significant differences in the contents of the analyzed flavonoids in the needles from *A. laricis*-infested twigs of *L. decidua* collected at different sampling times were found for the following compounds: flavanols catechin and epicatechin; flavanonols ampelopsin and taxifolin, and flavonols kaempferol and rutin (Figure 5a–c).

The lowest contents of catechin and epicatechin were noted in the needles collected on 20th of April. In the needles collected on 10th of May and 19th of June, the contents of both flavanols were significantly higher (2.7 and 3.0 times higher for catechin and 2.2 and 2.2 times higher for epicatechin, respectively) as compared to needles collected on 20th of April (Figure 5a).

The amounts of ampelopsin and taxifolin were the lowest in needles from 20th of April and the highest in needles from 10th of May (12.0 times higher for ampelopsin and 5.0 times higher for taxifolin) as compared to needles collected on 20th of April. In the needles collected on 19th of June, a statistically significant decrease in the content of these compounds was noted (1.5 times lower content for ampelopsin and 1.5 times lower for taxifolin) as compared to needles collected on 10th of May (Figure 5b).

The highest content of kaempferol was noted in needles collected on 20th of April. In the needles from 10th of May and 19th of June, the content was 3.0 and 5.5 times lower, as compared to needles collected on 20th of April, respectively. A significant increase in the content of rutin was noted in needles from 10th of May and 19th of June as compared to needles collected on 20th of April (1.4 and 1.6-fold increase, respectively) (Figure 5c).

### 3.4. Morphology and Anatomy of Larix decidua Needles Infested by Adelges laricis

**Figure 6:**
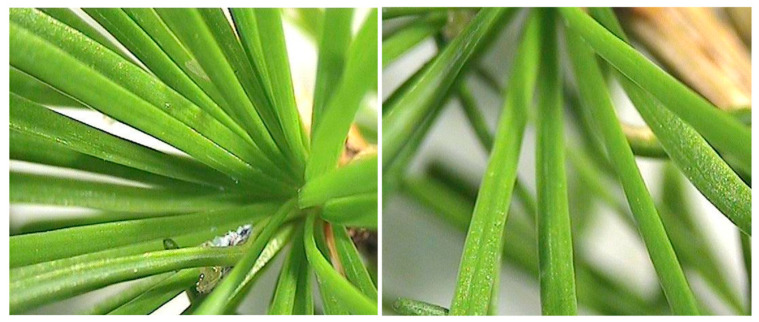
Healthy needles of *Larix decidua*.

**Figure 7:**
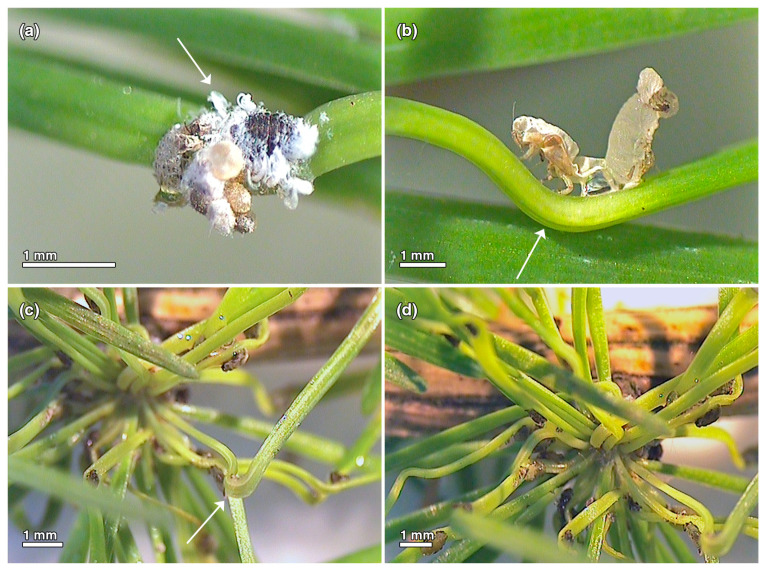
Morphological changes in *Larix decidua* needles after *Adelges laricis* infestation. (**a**) *Exulis progrediens* ‘wooly’ female with eggs and honeydew; the arrow points to the deformation of the needle at the feeding site of the adelgid. (**b**) Deformation and discoloration of the needle; the arrow points at the bend in the middle of the needle. In the photo, the *exuvium* of the crawler (**left**) and the late instar nymph (**right**) are visible. (**c**) Twisting and chlorosis of the needles after adelgid infestation; the arrow points at the bend in the middle of the needle. Winged females visible on the needles. (**d**) Twisting, crinkling, and chlorosis of the needles after adelgid infestation. Winged females visible on the needles.

The healthy uninfested needles of *L. decidua* are light green, 2–5 cm long, straight and slender, relatively soft (Figure 6). The adelgid-infested needles are curved, twisted, crinkled and discolored (Figure 7a–d). The deformations occur in the needles infested by the mobile nymphs of *A. laricis*, the crawlers (EPC) that hatch in the spring and search for a suitable place to settle (Figure 7b), as well as in the needles infested by the ‘woolly’ nymphs and ‘woolly’ adult females (EPW) that developed from the crawlers and remained at the place chosen by crawlers for the rest of their lives (Figure 7a). At the end of June, when adelgids migrate from the larch, the needles are entirely deformed and discolored (Figure 7c,d).

**Figure 8:**
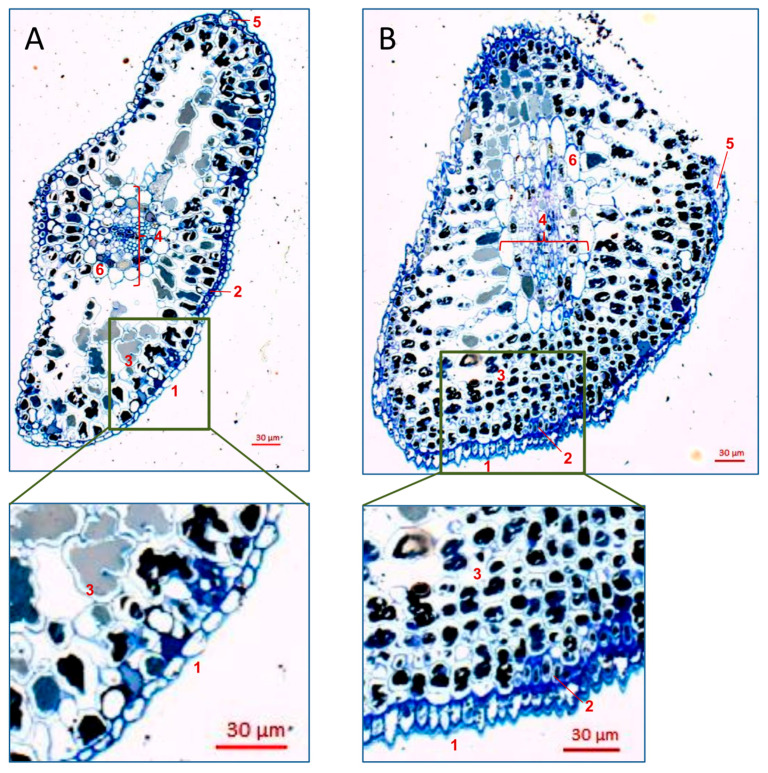
Cytomorphology of *Larix decidua* needles. (**A**) Uninfested (*Adelges laricis*-free) elliptical shaped needle; the enlargement shows one layer of thin-walled epidermal cells covered with thin layer of wax and mesophyll cells with highly wrinkled walls; (**B**) *Adelges laricis*-infested needle, flattened laterally; the enlargement shows one layer of cone-shaped epidermal cells, with the apex directed outwards and very thick layer of polar-arranged wax, and numerous mesophyll cells with poorly wrinkled walls. 1—epidermis; 2—hypodermis; 3—mesophyll; 4—vascular bundle; 5—resin duct, 6—endodermis.

Cytomorphological examination of *L. decidua* needles showed that the uninfested (adelgid-free) needles had an elliptical shape with evenly and tightly arranged epidermal cells covered by thick waxy cuticle. Below the epidermis, the hypodermis composed of diametrically layered sclerenchymatous cells with thick cell walls was observed. Mesophyll was formed by cells with highly wrinkled walls and with large light of cytosol. The cross-section also showed two small resin ducts placed just below the epidermis in lateral sides of the needle and the centrally located vascular bundle. The vascular system was protected by one-layered endodermis. The cross-section also showed small resin ducts placed to the marginal sides of the needle and the centrally located vascular bundle, protected by one-layered endodermis (Figure 8A).

The adelgid-infested needles showed cytomorphological deformations. The cross-section of the needle showed a lateral flattening. Tightly pressed together cells of epidermis, discontinuous hypodermis and a homogenous mesophyll surrounded the vascular bundles. The epidermis consisted of a single layer of cone-shaped cells, with the apex directed outwards. In addition, epidermal cells were covered by a very thick polar-arranged layer of wax. Below the epidermis, diametrically layered sclerenchymatous cells with thick cell walls (hypodermis) were observed occasionally. Mesophyll was formed by several layers of smaller cells, as compared to the mesophyll cells in uninfested needles, with poorly wrinkled walls but with persistent still large light of cytosol (Figure 8B).

## 4. Discussion

The appearance of an adelgid population often has a character of an invasion or an outbreak [20,21,22,24]. It was also the case in our study: the density of adelgids at the peak of population development was close to a hundred individuals per 25 cm twig, on average. The development of the larch wooly adelgid population on the European larch in our study was also typical for the species in the region. The timing of the appearance, peak and decline of the population occurred, respectively, at the end of April, in the beginning of May and at the end of June, as was reported in our other detailed populational study [70]. The rapid increase in population numbers results from the mass hatching of crawlers from eggs deposited by females the previous year. The period with a high number of adelgids lasted for approximately 2 weeks. Then, a rapid decline in population size followed, which might have resulted from the fact that a part of the population, namely the winged *sexupara* females, flew to the primary host, the spruce [70]. Moreover, the increase in rainfall in May and June as compared to April might have contributed to the decline in adelgid numbers. A similar pattern of population dynamics occurs in other adelgid species [74,75,76,77] or, more broadly, in other hemipteran species that need to alternate between primary and secondary hosts to complete their life cycle, e.g., the aphids [78,79,80]. The abiotic factors-temperature and rainfall are important variables for insect survival, behavior, abundance, and distribution, with temperature being the most dominant variable [81]. In aphids, the closest relatives of the adelgids, temperature is positively correlated with population growth rates through the season [82,83,84]. Dancewicz et al. [70] showed that the mean temperature had a significant negative effect on the number of *A. laricis exulis sistens* females at dwarf stem bases, but it was positively correlated with the number of winged *sexsuparae* females. Moreover, the most important limiting weather component was high rainfall, affecting mainly the crawlers [70].

Our study demonstrated that the heavy infestation of the larch tree, which was around 100 adelgids per twig at the peak of the population, caused profound changes in the infested organs of the larch tree—the needles—that might have been responses of the plant to the feeding by *A. laricis*. These changes involved both the structural and allelochemical traits of the needles.

The alterations in the structure of the infested needles due to *A. laricis* infestation were the deformations of the whole needles and aberrations in the morphology of internal tissues.

The whole-needle deformations were the most evident manifestations of *A. laricis* infestation and were expressed as twisting, folding, and discoloration and chlorosis. The twisting and folding of the leaf tissue are frequently reported symptoms of infestations by phytophagous hemipterans and may result from mechanical damage, perhaps due to the phytotoxicity of the saliva injected into plant tissues, or other physiological perturbations associated with mouthpart stylet penetration of plant tissues [85]. The discoloration observed in the adelgid-infested larch needles in the present study was probably a symptom of chlorosis, i.e., a general reduction in total chlorophyll and carotenoids, which is a frequently reported response to hemipteran feeding [86]. In aphids, the injection of saliva can lead to localized chlorosis near the feeding site and around the stylet tracks, caused by chloroplast disruption [87]. Our findings confirm the previous incidental reports on deformations and discolorations of needles after *A. laricis* feeding [11,20].

The histological alterations in the *A. laricis*-infested larch needles reported in the present study were associated with the shape, the number, and the extracellular depositions of cells composing different tissues, as compared to uninfested needles. First, the cells of the epidermis were narrower, more densely packed, and produced more wax on the outside. Second, the hypodermis was discontinuous and composed of only diametrically layered cell clusters. Third, the mesophyll cells were smaller and with smoother cell walls, but they were more numerous. The alterations in histological structure of plant organs are often responses to various abiotic and biotic stresses [33,88,89,90,91,92]. For example, an increase in mesophyll palisade tissue and the number of cell layers and a reduction of spongy tissue, cell volume, and cell space are adaptations of plants to drought conditions [93,94,95,96]. Meng et al. [91] showed that the cells in *Pinus sylvestris* L. (Pinaceae) needles were deformed under drought stress. The thickening of epidermal cuticle, the thinning of hypodermic cell walls, and the reduction of the phloem are attributed to anthropogenic pollution [89]. In *Larix sibirica* Ledeb. (Pinaceae), the increasing tendency in the thickness and area of the assimilation tissue in the needle occurred at low, medium and high levels of stand pollution [90]. Considering the biotic stresses, the physical and chemical characteristics of epidermis, such as thickness, cell arrangement, trichomes, and volatiles, can significantly affect host selection and colonization by herbivores, including aphids [97]. A study on epidermal cell structure showed that the resistance of sorghum to aphids was positively correlated with epidermal cell regularity and negatively correlated with the intercellular space and leaf thickness [98]. The strongly built-up layer of wax and lignin reduces the possibility of tissue penetration by pests, as well as effective infection by potential pathogens. It was demonstrated that the biomechanical properties of needle cushions in hemlocks *Tsuga* spp. (Pinaceae) may hinder colonization by crawlers of the hemlock woolly adelgid, *Adelges tsugae* (Annand) (Hemiptera: Adelgidae) and/or minimize the negative effects of adelgid feeding on the hemlock host due to greater difficulty in stylet penetration [99]. In the present study, we found that the strengthening of mechanical properties of needle tissues (e.g., more densely packed epidermal cells with a thicker cuticular wax layer) can be induced by the larch wooly adelgid crawlers’ feeding. Similar reactions of plants were also reported in other systems. For example, feeding by *Macrosiphum euphorbiae* (Thomas) (Hemiptera: Aphididae) caused mesophyll cell wall thickening in *Solanum tuberosum* L. (Solanaceae) [100]. In *Eucalyptus camaldulensis* Dehnh (Myrtaceae), feeding by Psyllidae and the concomitant secretion of toxic saliva caused ultrastructural changes interpreted as resembling cellular aging [92]. The cell wall modifications in response to aphid infestation may present an enhanced mechanical barrier to stylet penetration, or could potentially contribute to signaling pathways that activate plant defenses [101]. As the adelgids are very similar to aphids as far as host plant exploitation is concerned, it is likely that the structural transformations of tissues in the infested needles have a similar protective effect. In addition, it is worth stressing that the cytomorphological alterations were recorded in the adelgid-infested needles collected on 10th May, which was as soon as one month after the first adelgids had been observed on the larch tree and coincided with the maximum population size. Following that date, the decline in the population due to emigration of *A. laricis* from the larch started. The crawlers, which most likely induce the changes in larch needle anatomy, select a settling place for the successive developmental stages, including the adults that stay at that place for the rest of their lives and reproduce parthenogenetically there [70]. At each molting event, adelgids withdraw their stylets from plants and have to start penetration with the newly formed mouthparts without changing the location [70,102]. It is likely that the altered structure of the needles hinders the penetration of the tissues. In aphids, morphological and physiological changes in the host plant may trigger the appearance of the winged migrant morphs [103]. We observed that the first winged migrants of *A. laricis* appeared when the symptoms of needle deformations became noticeable. Considering these facts, it may be presumed that the alteration of the needle structure contributes to the decline in the larch wooly adelgid population on *L. decidua*. However, this hypothesis needs further, more focused studies.

Parallel to the changes in structure, the quantitative and qualitative changes in the allelochemistry of the larch needles occurred as a consequence of *A. laricis* infestation. Our study demonstrated that the content of the plant defense compounds in the needles from adelgid-infested twigs changed during the season, and the changes corresponded with the development of the *A. laricis* population on *L. decidua*. First, the total content of phenolic compounds doubled and second, the quantity of individual flavonoids increased or decreased according to the increase in the density of the larch wooly adelgid on the European larch.

Plant phenolics may be divided in two classes: preformed phenolics that are synthesized during the typical and regular development of plant tissues, and induced phenolics that are synthesized by plants in response to physical injury, infection by pathogens or attack by herbivores. The induced phenolics may also be constitutively present, but their synthesis is enhanced under abiotic or biotic stresses [46]. An increase in the concentration of phenolics in the needles of black pine *Pinus nigra* Ait. and Norway spruce *Picea abies* L. Karst (Pinaceae) was recorded in response to air pollution, an increased concentration of carbon dioxide, degree of solar radiation, and ozone exposure [104,105,106]. The constitutive as well as the induced content of phenolics may protect plants against herbivory, as was reported in many studies on different plant-herbivore systems [107]: the content of total phenolic compounds in the infested leaves of tolerant rice varieties was higher than that of susceptible rice varieties [108], the increased production of phenolics increased the resistance of the European silver birch *Betula pendula* Roth [104], and the higher content of phenolic compounds was associated with the resistance of *P. abies* to the eastern spruce gall aphid *Adelges abietis* L. [109]. The phenolic compounds can be used as predictors of Norway spruce resistance to the bark beetles (Coleoptera: Scolytidae) [110]. The combination of biotic and abiotic stresses may also affect the content of phenolics: under low light stress and aphid infestation, the level of phenolics drops significantly [73].

In the present study, the content of total phenols in larch needles was its lowest during the initial phase of adelgid infestation on 20th April, when only a few individuals on needles were observed. The amount of phenols recorded at that time reflected the constitutive level of these defense compounds. When the adelgid population reached its highest density 2 weeks later, on 10th May, the content of total phenols was twice as high as at the beginning of the season and continued to increase until the adelgid population decline on the larch tree. It is likely that the feeding activity of crawlers, the ‘woolly’ sessile nymphs and ‘woolly’ adults that predominated in the population at that time, caused an increase in the synthesis and accumulation of phenolic compounds in the larch needles. This strongly suggests that these compounds are involved in the defense system of the European larch against *A. laricis*. Our results also show that there is a variation in the contribution of individual compounds to this dynamic system. The selective induction of synthesis of defense compounds by the European larch in response to *A. laricis* infestation was found as well. The needles from adelgid-infested twigs of *L. decidua* contained the same nine flavonoids: the flavonols isorhamnetin, kaempferol, quercetin, and rutin, the flavanols catechin and epicatechin, the flavone apigenin, and flavanolols ampelopsin and taxifolin, regardless of the collection time and the phase in *A. laricis* population development. However, the content of individual flavonoids differed at different phases of *A. laricis* infestation. At the initial phase of adelgid infestation (20th April), the most abundant flavonoids in the needles were catechin, epicatechin, and kaempferol. At the peak in adelgid numbers (10th May), the most abundant were again catechin and epicatechin, whose contents had increased twofold as compared to the 20th April level. Of the remaining flavonoids, the amounts of ampelopsin and taxifolin were twelve and five times higher as compared to their initial amounts, respectively. In contrast, the amount of kaempferol was three times lower. The amounts of apigenin, rutin, isorhamnetin and quercetin did not change. At the population decline one month later (19th June), the catechin and epicatechin contents remained at high levels, and a slight, but not statistically significant, increase was observed. In contrast, although the contents of ampelopsin and taxifolin remained relatively high, their amounts were 1.5 and 1.5 times lower as compared to the previous measurements on 10th May, respectively. The contents of the remaining flavonoids remained constant at relatively low levels.

The relationship between plant susceptibility/resistance to herbivores and the content of flavonoids in plants is unquestionable and has been confirmed in every system studied [107]. At the same time, the involvement of individual compounds in plant defense is strongly herbivore species-specific, as was explicitly demonstrated in various aphid–flavonoid systems [111,112,113,114].

The increase in the content of catechin, epicatechin, ampelopsin and taxifolin in response to adelgid infestation found in the present study shows that in the *A. laricis—L. decidua* herbivore—plant defense system, these flavonoids play the major role. In general, catechins show diverse biological properties, including multiple effects on human health with antimicrobial, antiviral, and anti-aging activities, and also have important defensive functions against herbivores and pathogens [115]. In particular, catechin is reported as a crucial defense flavonoid in conifer trees [116,117,118]. For example, a strong increase in catechin content in the phloem of *P. abies* and *L. decidua* occurred after attack by *Ips typographus* L. and *I. cembrae* Heer (Coleoptera: Scolytidae) [10]. Moreover, the high concentration of catechin in the wound zone caused by bark beetles is considered the best predictor of *P. abies* resistance to these herbivores [110]. Ampelopsin and taxifolin are flavanonols whose biological activities include multiple roles, such as anti-bacterial, anti-cancer, antioxidant, hepatoprotective and anti-hypertension functions [119,120] as well as participation in plant–herbivore interactions [121]. In herbivore–plant interactions, taxifolin exhibits interesting properties. Apart from directly affecting herbivores [122,123], it inhibits detoxification enzymes and synergizes insecticides [124,125].

The present study analysis showed that ampelopsin and taxifolin occurred in several hundred times lower concentrations in the larch needles, as compared to catechin and epicatechin. Nevertheless, the increase in the content of these flavonoids due to adelgid infestation was noticeable. However, after the peak in adelgid infestation, a decrease in the content of both ampelopsin and taxifolin occurred. This phenomenon is probably associated with the fact that biosynthetically, these compounds share a common chalcone precursor and are therefore biogenetically and structurally related; both belong to flavanolols, the flavonoid subgroup [126,127]. In addition, ampelopsin (also known as dihydromyricetin) and taxifolin (also known as dihydroquercetin) are precursors of catechin, and catechin is a component of condensed tannins, the first-line defense chemicals of conifer trees [128,129,130,131,132]. The induced higher levels of catechin, epicatechin, ampelopsin and taxifolin in the adelgid-infested larch needles might therefore indicate that a process of increased condensed tannin synthesis was in progress. Such dependence was reported as a result of *Chaitophorus populeti* (Panzer) (Hemptera: Aphididae)–*Populus tremula* L. (Salicaceae) interaction [133]. In this context, the initial increase and then decrease in taxifolin and ampelopsin contents as well as a decrease in kaempferol amounts in the larch needles in relation to the development of adelgid infestation observed in the present study seems explainable. A significant decrease in kaempferol content occurred in the larch needles of *L. decidua* after the peak in adelgid infestation. The dynamic changes in the content of certain compounds may illustrate the fact that the plant under herbivore stress mobilized the available resources using the existing pool of preformed flavonoids to generate compounds with more potent activities, in this particular case, the condensed tannins. Such a strategy is in agreement with the concept of optimal use of resources and the flexibility in resource allocation during plant defense responses [133,134]. At the same time, independently from the utilization of the existing flavonoids, an intensification of flavonoid synthesis took place in response to damage caused by the adelgids. However, this idea needs further study to be confirmed in the *A. laricis–L. decidua* system.

An increase in the concentration of phenolics is often observed as a result of induction by attacking insects, which in turn may cause an increase in levels of plant resistance to herbivorous insects [107]. Simple phenols and polyphenols were studied in the foliage of *P. abies*, known to be either resistant or susceptible to *A. abietis*. Total phenol content was higher in resistant than in susceptible trees, and the presence of one unknown phenol compound and a higher total phenol content were considered associated with adelgid resistance in Norway spruce [109]. The induced response to adelgid fundatrices feeding on spruces is a rapid, hypersensitive reaction that does not have a delayed impact. Resistant tree genotypes have higher constitutive levels of phenolic compounds [109] that increase locally in the area surrounding the feeding site and are associated with the collapse and death of affected cells, whereas the feeding by *A. abietis* on susceptible trees induces galls [135]. Lattanzio et al. [113] identified a relationship between the content of flavonoids, namely quercetin, kaempferol and isoramnetin, and the level of susceptibility or resistance to the occurrence of and feeding by aphids: the tissues of more-resistant plants showed a higher content of flavonoids. Togola et al. [114], examining the resistance of various cowpea *Vigna unguiculata* (L.) Walp. (Fabaceae) lines to plant colonization by the cowpea aphid *Aphis craccivora* Koch, also showed the presence of quercetin and kaempferol in more-resistant plants. Moreover, a high concentrations of rutin [136,137,138,139] and kaempferol [113] were found in plant cultivars resistant to herbivorous insects. Kaempferol was detected only in the ‘Aldana’ cultivar of soybean *Glycine max* (L.) Merr, which was the soybean cultivar least acceptable to pea aphid *Acyrthosiphon pisum* (Harris), and might be responsible for the observed strong antixenosis resistance of this cultivar [111]. Infestation by cassava mealybug *Phenacoccus manihoti* Matile-Ferrero (Hemiptera: Pseudococcidae) was followed by an increase in the level of rutin [136,140]. Exposure to quercitin reduced the infestation of winter wheat *T. aestivum* by nymphs and apterous females of the bird cherry-oat aphid [141]. The quercitin concentration in plants increased in response to mango aphid *Toxoptera odinae* (Van der Goot) infestation of Chinese tallow *Tradica sebifera* (L.) Small (Euphorbiaceae) [142]. Among the flavonoids, the phytoalexin pisatin was observed to accumulate in high amounts in leaf cells of *Pisum sativum* L. after 48 h of colonization by pea aphids. Moreover, this increase correlated with the number of aphids colonizing the pea seedlings [52].

The localization of secondary metabolites within plant tissues has greater implications for resistance against phloem-feeding insects than herbivores exploiting other plant tissues. Phenolics are typically stored in vacuoles and/or specific storage tissues [106,143]. In addition, the glycoside forms of flavonoids may be diffused into the phloem [10,136,144] and xylem [145]. If phenolics are present mainly in the mesophyll, such compounds may be encountered during probing, resulting in the deterrence of phloem feeders like aphids and adelgids [13]. If localized in the phloem, nutritional deficiencies may occur, resulting in reduced developmental rates and/or fecundity [146]. The bird cherry-oat aphid *Aphis fabae* (Scopoli) exhibits reduced fecundity on cowpea *V. unguiculata* lines with elevated quercitin levels [113]. Exposure to quercitin increased the developmental time, the pre-reproductive period, and mortality, and decreased fecundity and the intrinsic rate of the natural increase in the pea aphid on an artificial diet [50].

In summary, the present study demonstrated that infestation of European larch by the larch wooly adelgid triggers two independent mechanisms of defense responses detectable in the attacked organs, the needles. One mechanism involves structural changes in the infested needles that probably impede adelgid stylet penetration in plant tissues, namely an increase in the number and degree of compression of epidermal cells and the increased production of the cuticular wax layer. The other mechanism involves dynamic changes in the defensive chemistry, namely an increased synthesis of specific flavonoids that are connected to condensed tannin synthesis. This study provides the first detailed information that can contribute to the understanding and practical application of defensive physical and/or chemical traits in the European larch against the larch wooly adelgid.

## Data Availability

The datasets used and/or analyzed during the current study are available from the corresponding author on reasonable request.

## Supplementary Materials

The following supporting information can be downloaded at: https://www.mdpi.com/article/10.3390/insects15120938/s1, Table S1. Flavonoids analyzed in needles from *Adelges laricis*-infested twigs of *Larix decidua* (μg/g dry weight; n = 3; mean ± SE; sample collection dates: 1–20 April, 2–20 May, 3–19 June).
