# Supplementary material for: Effects of Larch Woolly Adelgid Infestation on Morphological, Histological and Allelochemical Traits of European Larch Needles
Source: Insects. 2024 Nov 28;15(12):938. doi: 10.3390/insects15120938 (PMC11677814; doi:10.3390/insects15120938)
Supplement: Supplementary file 1 [file insects-15-00938-s001.zip › insects-3303849-supplementary.pdf]

Table S1. Flavonoids analysed in needles from *Adelges laricis*-infested twigs of *Larix decidua* (µg/g dry weight; n=3; mean ± SE).

Phenolic compounds	sample collection dates			P
	20 April	10 May	19 June	
Flavonols				
Isorhamnetin	18.03 ± 0.42	19.44 ± 1.72	16.81 ± 2.48	0.5962
Kaempferol	74.50 ± 8.21 a	25.11 ± 4.37 b	13.50 ± 1.14 b	0.0005
Quercetin	4.00 ± 0.27	3.43 ± 0.50	2.71 ± 0.30	0.1252
Rutin	3.34 ± 0.07 a	4.56 ± 0.14 b	5.37 ± 0.44 b	0.0054
Total	99.86 ± 8.20 a	52.55 ±6.40 b	38.39 ± 4.12 b	0.0013
Flavanols				
Catechin	259.72 ± 32.12 a	705.27 ± 104.86 b	778.89 ± 125.30 b	0.0176
Epicatechin	135.28 ± 9.25 a	298.06 ± 7.24b	303.33 ± 12.76b	0.0000
Total	395.00 ± 24.29 a	1003.33 ± 108.89 b	1082.22 ± 136.79 b	0.0059
Flavone				
Apigenin	1.57 ± 0.05	2.38 ± 0.36	2.54 ± 0.33	0.1005
Flavanonol				
Ampelopsin	1.64 ± 0.10 a	19.91 ± 1.82 b	12.89 ± 0.39 c	0.0001
Taxifolin	3.01 ± 0.04 a	15.08 ± 1.17 b	9.75 ± 0.29 c	0.0001
Total	4.65 ± 0.13 a	35.00 ± 0.02 b	22.64 ± 0.12 c	0.0000

Different letters in rows represent statistically significant differences among flavonoid concentration in needles from *Adelges laricis*-infested twigs of *Larix decidua* at P = 0.05 (ANOVA, post-hoc Newman–Keuls test).
